# Under-ascertainment of breast cancer susceptibility gene carriers in a cohort of New Zealand female breast cancer patients

**DOI:** 10.1007/s10549-020-05986-8

**Published:** 2020-10-28

**Authors:** Vanessa Lattimore, Michael T. Parsons, Amanda B. Spurdle, John Pearson, Klaus Lehnert, Jan Sullivan, Caroline Lintott, Suzannah Bawden, Helen Morrin, Bridget Robinson, Logan Walker

**Affiliations:** 1 Mackenzie Cancer Research Group, Department of Pathology and Biomedical Science, University of Otago, Christchurch, New Zealand; 2 Genetics and Computational Biology Division, QIMR Berghofer Medical Research Institute, Brisbane, QLD Australia; 3 Department of Pathology and Biomedical Science, University of Otago, Christchurch, New Zealand; 4 Centre for Brain Research and School of Biological Sciences, The University of Auckland, Auckland, New Zealand; 5 Genetic Health Service NZ, South Island Hub, Christchurch Hospital, Christchurch, New Zealand; 6 Cancer Society Tissue Bank, Department of Pathology and Biomedical Science, University of Otago, Christchurch, New Zealand; 7 Canterbury Regional Cancer and Haematology Service, Canterbury District Health Board, Christchurch Hospital, Christchurch, New Zealand

**Keywords:** Breast cancer, Panel gene, Variant classification, New Zealand

## Abstract

**Background:**

Diagnostic screening for pathogenic variants in breast cancer susceptibility genes, including *BRCA1*, *BRCA2, PALB2*, *PTEN* and *TP53*, may be offered to New Zealanders from suspected high-risk breast (and ovarian) cancer families. However, it is unknown how many high-risk pathogenic variant carriers in New Zealand are not offered genetic screening using existing triage tools and guidelines for breast (and ovarian) cancer patients.

**Methods:**

Panel-gene sequencing of the coding and non-coding regions of the *BRCA1* and *BRCA2* genes, and the coding regions and splice sites of *CDH1*, *PALB2*, *PTEN* and *TP53*, was undertaken for an unselected cohort of 367 female breast cancer patients. A total of 1685 variants were evaluated using the ENIGMA and the ACMG/AMP variant classification guidelines.

**Results:**

Our study identified that 13 (3.5%) breast cancer patients carried a pathogenic or likely pathogenic variant in *BRCA1*, *BRCA2, PALB2*, or *PTEN*. A significantly higher number of pathogenic variant carriers had grade 3 tumours (10/13) when compared to non-carriers; however, no other clinicopathological characteristics were found to be significantly different between (likely) pathogenic variant carriers and non-carriers, nor between variant of unknown significance carriers and non-carriers. Notably, 46% of the identified (likely) pathogenic variant carriers had not been referred for a genetic assessment and consideration of genetic testing.

**Conclusion:**

Our study shows a potential under-ascertainment of women carrying a (likely) pathogenic variant in a high-risk breast cancer susceptibility gene. These results suggest that further research into testing pathways for New Zealand breast cancer patients may be required to reduce the impact of hereditary cancer syndromes for these individuals and their families.

**Electronic supplementary material:**

The online version of this article (10.1007/s10549-020-05986-8) contains supplementary material, which is available to authorized users.

## Introduction

In New Zealand, over 3000 people are diagnosed with breast cancer each year [1]. The risk of breast cancer is significantly enhanced when an individual carries a disease-predisposing variant in high-risk cancer susceptibility genes, such as *BRCA1* and *BRCA2*, which, respectively, confer a 72% and 69% cumulative risk of breast cancer, and a 44% and 17% cumulative risk of ovarian cancer [2]. Identification of disease-associated genetic changes in breast cancer susceptibility genes, including *BRCA1*, *BRCA2* and *PALB2*, not only has actionable implications for carriers, but also for the additional members of their family who are found to carry the high-risk variant. Breast cancer prevention strategies, including surgical interventions (bilateral mastectomy) and prophylactic hormone therapy (tamoxifen and aromatase inhibitors), can help to mitigate the risk of developing the disease, while increased surveillance measures can aid with earlier detection. Identification of high-risk variants can also influence clinical decisions around patient treatment, including surgical options (i.e. choosing to have a total mastectomy instead of a wide local excision), while PARP inhibitors and cisplatin are key chemotherapeutic options for the treatment of cancers in patients carrying *BRCA1* and *BRCA2* pathogenic variants [3, 4].

Powerful new high-throughput DNA sequencing technology is now being adopted by diagnostic laboratories worldwide, enabling cheaper genetic testing across the entire gene(s) of interest for a greater number of individuals. Targeted multigene panel sequencing enables testing of genes known to be associated with familial breast cancer in addition to other known hereditary cancer syndromes, of which breast cancer is a component, namely, *TP53* (early onset breast cancer), *CDH1* (invasive lobular, hereditary diffuse gastric cancer syndrome) and *PTEN* (Cowden syndrome) [5–7]. Interpreting genetic data from gene tests can be challenging for health care providers, requiring a multidisciplinary collaboration of clinicians and scientists worldwide. The international ENIGMA (Evidence-based Network for the Interpretation of Germline Mutant Alleles) consortium [8] is an important initiative that was established to share expert advice for classification of *BRCA1* and *BRCA2* variants in databases, including ClinVar (https://www.ncbi.nlm.nih.gov/clinvar/) and BRCA Exchange (https://brcaexchange.org/). The ACMG/AMP (American College of Medical Genetics and Genomics (ACMG) and the Association for Molecular Pathology (AMP)) standards and guidelines provide an alternative approach for the interpretation of genetic variants associated with genetic diseases [9]. These guidelines describe a framework for classifying variants using multiple categories and degrees of evidence defined as very strong, strong, moderate or supporting.

Publicly funded gene screening is offered to New Zealand breast cancer patients by Genetic Health Service New Zealand (GHSNZ) if they fulfil testing criteria provided by eviQ Cancer Genetics (Breast and Ovarian Referral Guidelines—https://www.eviq.org.au/). Based on these guidelines, individuals may meet criteria based on their tumour pathology (negative status for oestrogen receptor, progesterone receptor and ERBB2/HER2 expression), or where a *BRCA1* and *BRCA2* pathogenic variant probability of 10% or more is calculated using a validated pathogenic variant prediction tool, such as the BOADICEA assessment programme [10]. Currently, approximately 9% of eligible New Zealand patients who have undergone genetic screening were found to carry a clinically actionable genetic variant in *BRCA1* or *BRCA2*. Many studies have shown that compared with multigene sequencing panels, testing of *BRCA1* and *BRCA2* alone ignores potentially actionable variants in a significant proportion of cases [11–13]. The panel of genes available for individuals who are offered gene screening through GHSNZ expanded in 2015 from *BRCA1* and *BRCA2*, to also include *PALB2* and *TP53*, and specific risk-associated variants in *CHEK2* and *ATM* (screening of *PTEN* and *CDH1* is offered if separate gene-specific criteria are met).

Early identification of carriers of high-risk variants in breast cancer susceptibility genes has the potential to reduce the number of breast and ovarian cancer cases and deaths in both the proband and their relatives. However, previous work has found that up to 50% of breast cancer patients who carry a pathogenic variant in a cancer-associated gene did not meet their national guidelines for genetic testing [14]. Recent studies from the United Kingdom and Australia have shown that multigene testing (*BRCA1*, *BRCA2* and *PALB2*, and *BRCA1* and *BRCA2*, respectively) for all breast cancer patients would be cost-effective when compared with current eligibility criteria based on personal and family-history testing [15, 16]. To date, the effectiveness of eligibility guidelines for genetic testing of breast cancer patients in New Zealand remains unclear.

This study used a gene panel sequencing approach to screen a consecutive series of unselected New Zealand female breast cancer patients for pathogenic variants in any of six high-risk breast cancer susceptibility genes (*BRCA1, BRCA2, CDH1, PALB2, PTEN* and *TP53*) and subsequently assessed the identified carriers for their eligibility for testing using national guidelines.

## Methods

### Patient cohort

The study cohort comprised 367 female breast cancer patients who underwent surgery at Christchurch Hospital (New Zealand) between May 2013 and April 2017. Informed, written consent was obtained for the tissue banking of pre-operative blood by the Christchurch Cancer Society Tissue Bank (CSTB) (HDEC 165TH92). Study-specific approval was obtained from the University of Otago Ethics Committee (H14/131) for this sequencing project. Extensively de-identified clinical and pathological characteristics were provided by the CSTB for all patients in the cohort, including additional family history and GHSNZ referral information for the patients identified to carry a (likely) pathogenic variant. Genetic screening results were ascertained for patients previously identified as eligible for publically funded gene screening by GHSNZ using the Breast and Ovarian Referral Guidelines eviQ (https://www.eviq.org.au/). This study was conducted in accordance with the CSTB guidelines, and under clinical guidance of Genetic Associates (JS, CL, SB) and a Senior Oncologist (BAR), thus providing an ethically approved process for actionable genomic variants to be returned to study participants.

### Molecular analysis

Germline DNA was purified from three punches of 3 mm diameter from blood stored on Flinders Technology Associates (FTA) cards (Whatman) using QIAamp DNA Investigator kit (Qiagen), following the manufacturer’s instructions. Nineteen of the initial 367 samples obtained from the Cancer Society Tissue Bank returned a yield of less than 15 ng of genomic DNA and were excluded from further analysis. Library preparation (Ion AmpliSeq Library Kit 2.0 (ThermoFisher Scientific)) and sequencing (Ion Torrent) using a custom gene panel were completed by the Liggins Institute (Auckland, New Zealand). The custom panel was designed using Ion AmpliSeq designer software (Life Technologies) to target the exons and introns of *BRCA1* and *BRCA2*, and the exonic regions of *CDH1*, *PALB2*, *PTEN* and *TP53*.

### Variant classification

Variants were numbered in accordance to HGVS recommendations (https://varnomen.hgvs.org), where + 1 represents the first nucleotide of the ATG translation initiation codon. Reference transcripts for variant annotation were as follows: NM_007294.3 (*BRCA1*); NM_000059.3 (*BRCA2*); NM_004360 (*CDH1*), NM_024675 (*PALB2*), NM_000314.6 (*PTEN*) and NM_000546.5 (*TP53*). Variant calls were processed using Ion Reporter Software, then annotated with VEP (v92.3) [17], MaxEntScan splicing predictions [18], ClinVar annotation (2018-05 full release, processed using the MacArthur Lab ClinVar processing pipeline [19]) and REVEL pathogenicity predictions [20]. *BRCA1* and *BRCA2* variants were assessed using the ENIGMA classification guidelines (v2.5.1) (Supplementary Fig. 1) and ACMG/AMP guidelines [9]. *CDH1*, *PALB2*, *PTEN* and *TP53* variants were classified using the ACMG/AMP guidelines, including the gene-specific guidelines for *CDH1* and *PTEN* variants [9, 21, 22]. Variants were classified into one of five categories: Pathogenic; Likely Pathogenic; Variant of Unknown Significance; Likely Benign; or Benign. (Likely) pathogenic variants were confirmed by sequencing PCR fragments by capillary electrophoresis using a 3730xl DNA Analyzer (Applied Biosystems). PCR primer sequences are listed in Supplementary Table 1.

## Results

Results from gene screening (*BRCA1*, *BRCA2*, *CDH1*, *PALB2*, *PTEN* and *TP53*) germline variants in 367 New Zealand female breast cancer patients identified 1,685 single-nucleotide variants or indels. Sanger validated thirteen of the thirty variants that were classified as (likely) pathogenic, whereas the remaining seventeen variants were identified as false-positive calls (Supplementary Table 2). Of the validated variants, ten women carried a high-risk (likely) pathogenic variant in *BRCA2*, and three women carried one (likely) pathogenic variant in *BRCA1*, *PTEN* or *PALB2*, respectively (Table 1). Seven of these were frameshift variants, four were nonsense variants and two were donor site variants. Of the nineteen false calls, nine were located in homopolymer regions. The average minor allele frequency was 0.20 for all of the false calls and 0.49 for all of the true calls.

**Table 1:** Pathogenic variants identified in New Zealand breast cancer patients

Gene	Variant	Amino acid chance (HGVS Protein)	Classification	Patient ID	Age diagnosed (years)	Proband cancer diagnosis	Referred for genetic screening
*BRCA2*	c.631 + 2 T > G		Likely Pathogenic	169	58	Invasive Ductal Carcinoma, NST	Referred
*BRCA2*	c.755_758del	p.(Asp252ValfsTer24)	Pathogenic	67	52	Invasive Ductal Carcinoma, NST	Not referred
*BRCA2*	c.2808_2811del	p.(Ala938ProfsTer21)	Pathogenic	186	63	Invasive Ductal Carcinoma, NST	Referred
*BRCA1*	c.5503C > T	p.(Arg1835Ter)	Pathogenic	242	81	Invasive Ductal Carcinoma, NST	Referred
*BRCA2*	c.4876_4877del	p.(Asn1626SerfsTer12)	Pathogenic	158	74	Invasive Ductal Carcinoma, NST	Not referred
*BRCA2*	c.5350_5351del	p.(Asn1784HisfsTer2)	Pathogenic	213	68	Invasive Ductal Carcinoma, NST	Not referred
*BRCA2*	c.4405_4409del	p.(Asp1469LysfsTer11)	Pathogenic	245	75	Invasive Ductal Carcinoma. NST	Not referred
*BRCA2*	c.4478_4481del	p.(Glu1493ValfsTer10)	Pathogenic	189	36	Invasive Ductal Carcinoma, NST	Not referred
*BRCA2*	c.7757G > A	p.(Trp2586Ter)	Pathogenic	50	62	Invasive Ductal Carcinoma, NST	Referred
*BRCA2*	c.5682C > G	p.(Tyr1894Ter)	Pathogenic	216	51	Invasive Ductal Carcinoma, NST	Referred
*BRCA2*	c.7007 + 1G > A		Likely Pathogenic	390	40	Invasive Ductal Carcinoma, Basal Phenotype	Referred
*PALB2*	c.196C > T	p.(Gln66Ter)	Pathogenic	77	71	Invasive Ductal Carcinoma, NST	Not referred
*PTEN*	c.406dup	p.(Cys136LeufsTer44)	Pathogenic	225	31	Invasive Carcinoma, apocrine	Referred

Referral to GHSNZ was recorded in the clinical notes of five of the ten patients confirmed to carry (likely) pathogenic variants in *BRCA2* and for the two patients confirmed to carry a pathogenic variant in *BRCA1* and *PTEN*, respectively (Table 1). Four of these seven referred patients had been tested for a known familial variant, whereas exploratory gene screening was undertaken for three patients.

The *PALB2* carrier and five of the *BRCA2* pathogenic variant carriers identified in this cohort had not been referred to GHSNZ. There was sufficient information in the hospital records for only one of these patients (ID 189) to establish that they would have met testing criteria. While this patient reported no known family cancer history, her age at diagnosis (36 y/o) alone meets eviQ criteria for referral (referral criteria: breast cancer < 40 years of age). The records for only one additional patient documented that family history was discussed at all during their treatment (ID 213), with one case of bowel cancer noted in their extended family.

Two of the non-referred patients (IDs 189 and 245) were from high-risk families known to carry a pathogenic variant. It is unknown whether these patients had chosen not to have predictive testing, or if they were unaware of their respective family histories. However, as noted above, patient ID 189 met criteria for referral based on her age at diagnosis alone. There was no indication that family history had been discussed for one of these patients (ID 245), whose age alone (74 years at diagnosis) may have influenced the clinician’s decision around GHSNZ referral.

A novel likely pathogenic variant in *PTEN* was identified in one patient, who was 31 years old at diagnosis and met eviQ criteria for referral (referral criteria: breast cancer < 40 years of age). This patient has a family history of breast cancer, but it was unknown whether she had clinical features or a family history indicative of *PTEN* hamartoma tumour syndrome. The *PTEN* variant (c.406dup) is predicted to cause a termination codon 44 amino acids downstream of the variant site, completely removing the crucial C2 and C-Terminal Tail domains of the 403-amino acid protein (Supplementary Fig. 2).

*PALB2* and *PTEN* screening was not routinely offered at the time each of the two patients found to carry a variant in one of these genes (IDs 77 and 225) presented with breast cancer. The *PTEN* variant carrier was eligible for *BRCA1* and *BRCA2* screening and was identified to carry the *BRCA1*[c.594-2A > C_c.641A > G] haplotype, which is now known to be benign [23].

Forty-one variants of unknown significance were identified in 35 (9.8%) women (Supplementary Table 3). Twenty of these variants were identified in *BRCA2*, nine in *BRCA1*, two in *CDH1*, eight in *PALB2* and two in *TP53*. The majority were missense (*n* = 34), while the remainder included four intronic variants and three variants located near a splice site (within 6 nt). Within our study cohort, 319 (86.9%) women were not found to carry any VUS or (likely) pathogenic variants (Table 2).

**Table 2:** Characteristics of study cohort separated by variant status

	Class 4/5	Class 3 (VUS)	Non-variant carriers	Class 4/5 OR [95% CI] P	Class 3 (VUS) OR [95% CI] P
Study cohort	13	35	319		
Age diagnosed					
< 50 years	3	10	64		
> 50 years	10	25	255	1.19 [0.21, 4.82] 0.73	1.13 [0.47, 2.51] 0.70
Ethnicity					
Maori	0	4	18		
Non-Maori	12	31	281		2.01 [0.47, 6.65] 0.27
Grade					
1	0	4	40		
2	3	12	129		
3	10	19	150	3.62 [1.06,17.18] 0.05	1.34 [0.63, 2.89] 0.48
Estrogen receptor					
Pos	10	28	266		
Neg	3	7	50	0.63 [0.15, 3.67] 0.45	0.75 [0.30, 2.15] 0.48
Progesterone Receptor					
Pos	8	24	239		
Neg	5	11	75	0.50 [0.14, 2.02] 0.32	0.69 [0.31, 1.62] 0.31
HER2					
Pos	0	3	45		
Neg	12	26	202		0.52 [0.10, 1.81] 0.44
Equivocal	1	3	30		
Tubules					
1	0	2	20		
2	2	10	81		0.69 [0.48, 2.67] 0.69
3	11	23	218		0.88 [0.41, 2.04] 0.85
Pleomorphism					
1	0	1	5		
2	0	10	113		0.73 [0.30, 1.64] 0.46
3	13	24	201		1.28 [0.58, 3.00] 0.58
Mitoses					
1	2	13	140		
2	3	7	68	1.11 [0.19, 4.46] 1.00	0.92 [0.32, 2.28] 1.00
3	8	15	111	2.99 [0.82, 11.89] 0.07	1.40 [0.64, 3.01] 0.36

Counts of samples in each category with odds ratios, 95% confidence intervals and Fisher exact tests between carriers and non-carriers with at least 1 observation in each cell. Grade has been collapsed to grade 3 vs grades 1 or 2

The majority of the (likely) pathogenic variant carriers had grade 3 tumours (10/13), which was significantly higher compared to non-carriers (*P* < 0.05). No other clinical characteristics were significantly different between (likely) pathogenic carriers and non-carriers, or between VUS carriers and non-carriers (Table 2). Of the patients in our cohort who specified their ethnicity, 7.1% (23/322) identified as Māori or as having Māori ancestry (Table 2). On average, Māori were found to be slightly younger at diagnosis compared to non-Māori (57.6 vs 63.1 years), and a higher proportion were diagnosed with grade 3 tumours compared to non-Māori (60.9% vs 47.8%) (Supplementary Table 4), but these clinical differences did not reach statistical significance (*P* < 0.05).

## Discussion

This study screened 367 female breast cancer patients in New Zealand for risk-associated variants in the familial breast cancer susceptibility genes *BRCA1*, *BRCA2*, *CDH1*, *PALB2*, *PTEN* and *TP53*. Thirteen patients (3.5%) were identified to carry (likely) pathogenic variants: ten in *BRCA2* and one in *BRCA1*, *PALB2* and *PTEN*, respectively.

*BRCA1*/*2* (likely) pathogenic variants were identified in 3.0% (11/367) of the cohort, which is within the range of observations in comparable international studies (1.8–5.4%) [24–27]. An additional two likely pathogenic variants (0.5%) were identified in *PALB2* and *PTEN.* (Likely) pathogenic variants in non-*BRCA1/2* breast cancer susceptibility genes (*CDH1*, *PALB2*, *PTEN* and *TP53*) have typically been observed in small numbers in other studies. *PALB2* and *PTEN* pathogenic variants were detected in 0.2% of a US breast cancer cohort analysed by Tung et al. [28]. In a German cohort of 581 breast cancer patients with a strong family history of breast/ovarian cancers, pathogenic variants in *PALB2*, *TP53* and *CDH1* were only found in 1%, 0.3% and 0% of the patients, respectively [29]. These results indicate that pathogenic variants in these genes are rare, even in very select high-risk populations. Assessing variants across all genes (*BRCA1*, *BRCA2*, *CDH1*, *PALB2*, *PTEN* and *TP53*) in our study also found 11.7% of New Zealand patients carrying a variant of unknown significance, which was comparable to similar international breast cancer studies (6.4% [30]; 9.9% [27]; 14.7% [31]).

Six of the 13 (likely) pathogenic variant carriers identified in our cohort were not referred to GHSNZ and there was sufficient information in the clinical notes for only one of these patients to suggest that they did meet the criteria for referral. Extrapolating this result nationwide suggests that around 2% of the approximately 3000 breast cancer cases diagnosed annually may carry a (likely) pathogenic variant in a susceptibility gene that predisposes to cancer, but would fail to be referred to GHSNZ. The issues that may prevent referral include that patients may not always be aware of their family cancer history, and/or of genetic testing in their extended families, as evidenced by two of the high-risk variant carriers identified in our study. Furthermore, one high-risk pathogenic variant carrier identified in our study (ID 67) refused all treatment options at the time of diagnosis, which may have affected referral to GHSNZ, while acceptable treatment options were explored. Due to this, clinicians may not be able to obtain the information required to establish if a patient is eligible for genetic referral, while in some cases the patient may not have been asked for this information [32]. These issues would each have direct implications for risk management advice offered to patients and their families.

The gene panel offered by GHSNZ since 2015 was not available when the *PALB2* and *PTEN* variant carriers identified in this work presented with breast cancer. While the more recent implementation of panel screening includes *PALB2* (in addition to *TP53* and specific variants in *ATM* and *CHEK2*), separate gene-specific criteria need to be met to also include *PTEN*. In addition, re-testing would need to have been offered, or exploratory testing in a close relative undertaken, to identify non-*BRCA1*/*2* disease-predisposing variants in high-risk patients tested prior to 2015.

Universal gene screening of breast cancer patients has been previously reported as a cost-effective option to overcome the under-referral of breast cancer patients who do not meet the current referral guidelines [16, 33]. Implementation of such a regime in New Zealand would overcome the factors identified in this study that limited patient referral, while simultaneously capturing many of the families that carry (likely) pathogenic variants in non-*BRCA1*/*2* genes that were not included in screening at the time of their referral. This strategy would improve identification of high-risk families in New Zealand, while the information gained would also help to guide the treatment plans of affected patients and aid with decisions around prevention for the other identified carriers in each patient’s family.

The clinical characteristics of all breast cancer cases reported by two northern New Zealand regional registries over a 13-year period have been summarized previously [34]. When comparing the clinical features of the two cohorts, our cohort reported a higher proportion of women presenting with a grade 3 tumour (48.8% vs 29.1%), fewer women under 50 years of age (21.0% vs 28.2%) and a lower number of women of Māori ethnicity (6.4% vs 9.5%). The latter finding reflects the population demographic differences between the North Island and the South Island of New Zealand, with higher proportion of people reporting Māori descent in the North Island. Four VUS carriers, but none of the (likely) pathogenic variant carriers, identified in our cohort reported to be of Māori decent. A novel pathogenic variant was identified in one patient in our study suggesting that there may be other unique high-risk variants in the New Zealand population. Future work to expand the cohort to include patients from the North Island would help us better understand the genetic predisposition to cancer in Māori, while also further capturing the genetic diversity of the New Zealand population.

This study is the first to demonstrate the frequency of (likely) pathogenic genetic variants in *BRCA1*, *BRCA2*, *TP53*, *CDH1*, *PTEN* and *PALB2* in a cohort of unselected breast cancer patients in New Zealand. Furthermore, we provide evidence that a significant proportion of (likely) pathogenic variant carriers are not being referred to a clinical genetic service, even when some of these patients were eligible under the current referral guidelines. Variant screening in all women diagnosed with breast cancer has been shown to be cost-effective, while reducing the risk that variant carriers remain unidentified [16, 33]. Gene panel testing of all breast cancer patients in New Zealand would improve the identification of high-risk individuals, allowing for timely predictive testing and appropriate risk management, while also reducing the impact of hereditary cancer syndromes for these individuals and their families.

## Electronic supplementary material

Below is the link to the electronic supplementary material.

Supplementary file1 (DOCX 290 kb)
