# Supplementary material for: Under-ascertainment of breast cancer susceptibility gene carriers in a cohort of New Zealand female breast cancer patients
Source: Breast Cancer Res Treat. 2020 Oct 28;185(3):583–90. doi: 10.1007/s10549-020-05986-8 (PMC7921023; doi:10.1007/s10549-020-05986-8)
Supplement: Supplementary file 1 — Supplementary file1 (DOCX 290 kb) [file 10549_2020_5986_MOESM1_ESM.docx]

**Under-ascertainment of breast cancer susceptibility genes carriers in a cohort of New Zealand female breast cancer patients.**

**Journal: Breast Cancer Research and Treatment**

Vanessa Lattimore^1^

Michael T. Parsons^2^

Amanda B. Spurdle^2^

John Pearson^2^

^\^Klaus Lehnert^4^

Jan Sullivan^5^

Caroline Lintott^5^

Suzannah Bawden^5^

Helen Morrin^1,6^

Bridget Robinson^1,7^

Logan Walker ^1^

*^1^Mackenzie Cancer Research Group, Department of Pathology and Biomedical Science, University of Otago, Christchurch, New Zealand*

*^2^Genetics and Computational Biology Division, QIMR Berghofer Medical Research Institute, Brisbane, Queensland, Australia*

*^3^Department of Pathology and Biomedical Science, University of Otago, Christchurch, New Zealand*

*^4^Centre for Brain Research and School of Biological Sciences, The University of Auckland, New Zealand.*

*^5^Genetic Health Service NZ, South Island Hub, Christchurch Hospital, New Zealand.*

*^6^Cancer Society Tissue Bank, Department of Pathology and Biomedical Science University of Otago, , Christchurch, New Zealand*

*^7^Canterbury Regional Cancer and Haematology Service, Canterbury District Health Board, Christchurch Hospital, Christchurch, New Zealand.*

Corresponding author:

Dr Vanessa Lattimore

*Mackenzie Cancer Research Group, Department of Pathology and Biomedical Science, University of Otago, Christchurch, New Zealand*

vanessa.lattimore@otago.ac.nz

Phone: +6433640557


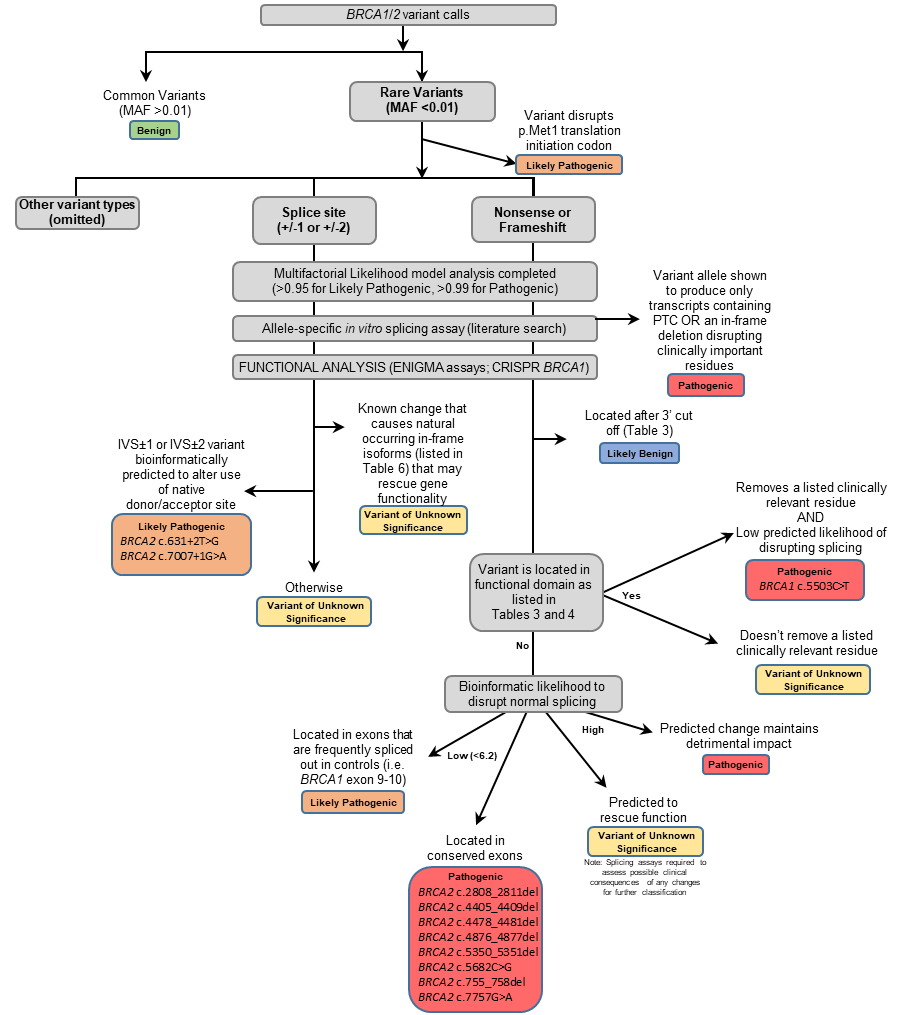


Supplementary Figure 1. Schematic summarising the ENIGMA multifactorial classification guidelines used to classify the (likely) pathogenic *BRCA1* and *BRCA2* variants associated with breast and ovarian cancers identified in this study. All missense, synonymous and deep intronic variants identified in this work were classified as either (Likely) Benign or Variant of Unknown Significance.


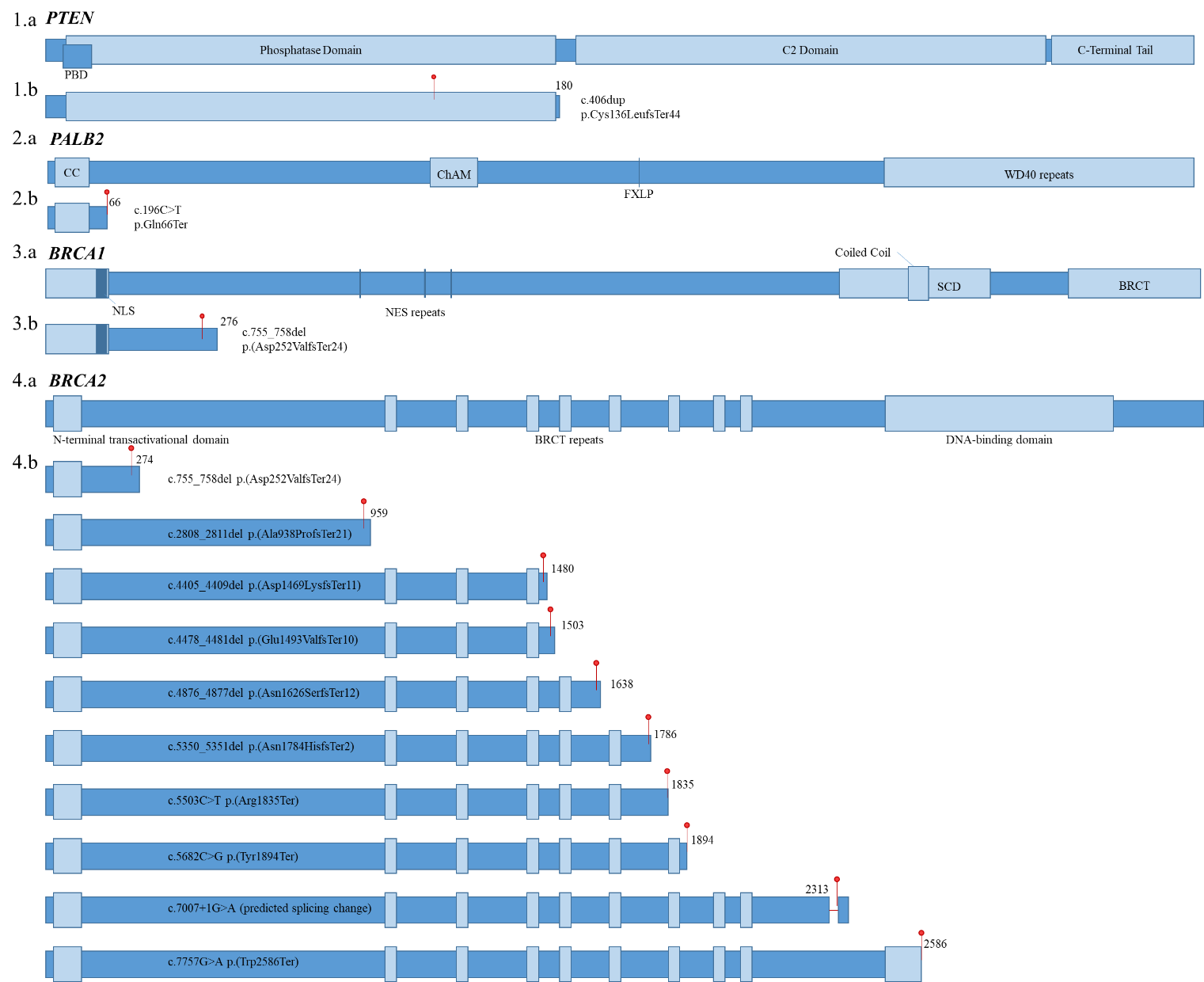
Supplementary Figure 2. Predicted mRNA transcript changes in the presence of high-risk variants. Full length mRNA transcripts of each gene (#.a) with the predicted mRNA transcripts of each (likely) pathogenic variant identified in this cohort shown below (#.b). Numbers represent the final transcribed amino acid in the transcript. Domain locations indicated by light blue boxes.

Abbreviations: CC, Coiled coil; ChAM, Chromatin-association motif; PBD, PIP2 binding motif.

Supplementary Table 1. Primer sequences for validating high-risk variant calls.

| Gene | Variant | Forward Primer | TM | Reverse Primer | TM | Amplicon Length |
| --- | --- | --- | --- | --- | --- | --- |
| *BRCA1* | c.550dup | CCCATGCCTTTAACCACTTC | 59.43 | TGCACATACATCCCTGAACC | 59.37 | 349 |
| *BRCA1* | c.2869C>T | ATCACTGCAGGCTTTCCTGT | 59.87 | CGGCTAATTGTGCTCACTGT | 58.95 | 311 |
| *BRCA1* | c.5503C>T | GATTGCGCCATCACACTCTA | 59.83 | AAGCTCATTCTTGGGGTCCT | 60.07 | 360 |
| *BRCA2* | c.24_25insA | GTTCCAGGAGATGGGACTGA | 60.05 | GGTTCTAAGCAACACTGTGACG | 59.84 | 328 |
| *BRCA2* | c.36del* | GTTCCAGGAGATGGGACTGA | 60.05 | GGTTCTAAGCAACACTGTGACG | 59.84 | 328 |
| *BRCA2* | c.631+2T>G | CGTTAAGTGAAATAAAGAGTGAATGAA | 59.26 | TGACAATTATCAACCTCATCTGC | 59.1 | 261 |
| *BRCA2* | c.755_758del | TTGGACCTAGGTTGATTGCAG | 60.12 | GGGTGACAGAGCAAGACTCC | 59.84 | 325 |
| *BRCA2* | c.1813del* | TTTGCTCACAGAAGGAGGACT | 59.08 | AAAAACACAGAAGGAATCGTCA | 58.74 | 346 |
| *BRCA2* | c.2588del | TCCCATGGAAAAGAATCAAGA | 59.49 | TGGGTTCGTTTACACAAGTCAA | 60.43 | 323 |
| *BRCA2* | c.2808_2811del | CTGTCAATCCAGACTCTGAAGAA | 58.59 | TTCAAGGAGATGTCCGATTTT | 58.63 | 313 |
| *BRCA2* | c.4284del* | CTGCTACTAAAACGGAGCAAAA | 58.76 | GTTGTCCCTGGAAGGTCACT | 59 | 300 |
| *BRCA2* | c.4361_4362insGA | CTGCTACTAAAACGGAGCAAAA | 58.76 | GTTGTCCCTGGAAGGTCACT | 59 | 300 |
| *BRCA2* | c.4405_4409del | CTGCTACTAAAACGGAGCAAAA | 58.76 | GTTGTCCCTGGAAGGTCACT | 59 | 300 |
| *BRCA2* | c.4478_4481del | CTGCTACTAAAACGGAGCAAAA | 58.76 | GTTGTCCCTGGAAGGTCACT | 59 | 300 |
| *BRCA2* | c.4876_4877del | GCTGCCCCAAAGTGTAAAGA | 60.25 | TTTTGCTTCAAGTAATGAAGTCTGA | 59.5 | 312 |
| *BRCA2* | c.5297del* | GACAAAAATCATCTCTCCGAAAA | 59.6 | AGGTGGCCCTACCTCAAAAT | 59.83 | 345 |
| *BRCA2* | c.5350_5351del | GACAAAAATCATCTCTCCGAAAA | 59.6 | AGGTGGCCCTACCTCAAAAT | 59.83 | 345 |
| *BRCA2* | c.5682C>G | GGCCACCTGCATTTAGGATA | 59.92 | TTCCAAACTAACATCACAAGGTG | 59.05 | 341 |
| *BRCA2* | c.7007+1G>A | CATTCACTGAAAATTGTAAAGCCTA | 58.85 | TCATTTATAAAAACGAGACTTTTCTCA | 58.93 | 251 |
| *BRCA2* | c.7667del | CGTATGGCGTTTCTAAACATTG | 59.55 | CAGAATGCTTAACCATAATGCAC | 58.71 | 330 |
| *BRCA2* | c.7757G>A | CGTATGGCGTTTCTAAACATTG | 59.55 | CAGAATGCTTAACCATAATGCAC | 58.71 | 330 |
| *BRCA2* | c.8940del | TTGTTCTGATTGCTTTTTATTCCA | 60.01 | GGGCATTAGTAGTGGATTTTGC | 59.87 | 375 |
| *CDH1* | c.164-1G>T | TCTTCCCACAAGTTCGCTCT | 59.99 | ACGGTACCAAGGCTGAGAAA | 59.73 | 389 |
| *PALB2* | c.196C>T | TTTCTGGGGCTGTTTTTGTC | 60.09 | CCAGCCTGGGTAACAAAGAG | 59.73 | 308 |
| *PALB2* | c.839del* | TTTTGGTTTTCATTTGCTGGT | 59.48 | ATTTCACCAGGGCGACTACA | 60.52 | 310 |
| *PTEN* | c.406dup | TTTGAAGACCATAACCCACCA | 60.21 | GAAACCCAAAATCTGTTTTCCA | 60.2 | 300 |
| *PTEN* | c.821del* | TCAGATTGCCTTATAATAGTCTTTGTG | 59.24 | TCTGCACGCTCTATACTGCAA | 59.79 | 310 |
| *TP53* | c.832C>G | TGCTAGGAAAGAGGCAAGGA | 60.09 | CAAGGGTGGTTGGGAGTAGA | 59.96 | 331 |

Supplementary Table 2. List of all sanger-sequence validated and false positive variants annotated as (likely) pathogenic.

| Sample ID | True/False call | Gene | Variant | Classification^b^ | Read count (WT, Alt) | % Alt allele |
| --- | --- | --- | --- | --- | --- | --- |
| 242 | TRUE | *BRCA1* | c.5503C>T | Pathogenic | 1961, 1829 | 48% |
| 67 | TRUE | *BRCA2* | c.755_758del | Pathogenic | 684, 671 | 50% |
| 189 | TRUE | *BRCA2* | c.4478_4481del | Pathogenic | 125, 121 | 49% |
| 213 | TRUE | *BRCA2* | c.5350_5351del | Pathogenic | 332, 401 | 55% |
| 245 | TRUE | *BRCA2* | c.4405_4409del | Pathogenic | 673, 528 | 44% |
| 50 | TRUE | *BRCA2* | c.7757G>A | Pathogenic | 1095, 932 | 46% |
| 186 | TRUE | *BRCA2* | c.2808_2811del | Pathogenic | 970, 1047 | 52% |
| 216 | TRUE | *BRCA2* | c.5682C>G | Pathogenic | 411, 412 | 50% |
| 77 | TRUE | *PALB2* | c.196C>T | Likely Pathogenic | 607, 536 | 47% |
| 390 | TRUE | *BRCA2* | c.7007+1G>A | Likely Pathogenic | 597, 523 | 47% |
| 158 | TRUE | *BRCA2* | c.4876_4877del | Pathogenic | 196, 199 | 50% |
| 169 | TRUE | *BRCA2* | c.631+2T>G | Likely Pathogenic | 1069, 1122 | 51% |
| 225 | TRUE | *PTEN* | c.406dup | Pathogenic | 684, 686 | 50% |
|  |  |  |  |  | **Average MAF:** | **49%** |
| 15 | FALSE | *BRCA1* | c.213-15A>G^a^ | Likely Pathogenic | 527,99 | 16% |
| 17 | FALSE | *BRCA1* | c.213-15A>G^a^ | Likely Pathogenic | 442,88 | 17% |
| 21 | FALSE | *BRCA1* | c.213-15A>G^a^ | Likely Pathogenic | 705,114 | 14% |
| 26 | FALSE | *BRCA1* | c.213-15A>G^a^ | Likely Pathogenic | 647,94 | 13% |
| 33 | FALSE | *BRCA1* | c.213-15A>G^a^ | Likely Pathogenic | 680,108 | 14% |
| 34 | FALSE | *BRCA1* | c.213-15A>G^a^ | Likely Pathogenic | 612,108 | 15% |
| 36 | FALSE | *BRCA1* | c.213-15A>G^a^ | Likely Pathogenic | 405,59 | 13% |
| 59 | FALSE | *BRCA1* | c.213-15A>G^a^ | Likely Pathogenic | 579,110 | 16% |
| 61 | FALSE | *BRCA1* | c.213-15A>G^a^ | Likely Pathogenic | 619,89 | 13% |
| 78 | FALSE | *BRCA1* | c.213-15A>G^a^ | Likely Pathogenic | 592,134 | 18% |
| 91 | FALSE | *BRCA1* | c.213-15A>G^a^ | Likely Pathogenic | 698,121 | 15% |
| 92 | FALSE | *BRCA1* | c.213-15A>G^a^ | Likely Pathogenic | 743,105 | 12% |
| 95 | FALSE | *BRCA1* | c.213-15A>G^a^ | Likely Pathogenic | 523,82 | 14% |
| 121 | FALSE | *BRCA1* | c.213-15A>G^a^ | Likely Pathogenic | 807,113 | 12% |
| 122 | FALSE | *BRCA1* | c.213-15A>G^a^ | Likely Pathogenic | 782,145 | 16% |
| 291 | FALSE | *BRCA1* | c.213-15A>G^a^ | Likely Pathogenic | 508,73 | 13% |
| 308 | FALSE | *BRCA1* | c.213-15A>G^a^ | Likely Pathogenic | 250,48 | 16% |
| 334 | FALSE | *BRCA1* | c.213-15A>G^a^ | Likely Pathogenic | 459,74 | 14% |
| 353 | FALSE | *BRCA1* | c.213-15A>G^a^ | Likely Pathogenic | 284,43 | 13% |
| 356 | FALSE | *BRCA1* | c.213-15A>G^a^ | Likely Pathogenic | 249,44 | 15% |
| 168 | FALSE | *BRCA1* | c.2869C>T | Pathogenic | 7,2 | 22% |
| 211 | FALSE | *BRCA1* | c.550dup | Pathogenic | 12,4 | 25% |
| 55 | FALSE | *BRCA2* | c.1813del^a^ | Pathogenic | 4,2 | 33% |
| 348 | FALSE | *BRCA2* | c.1813del^a^ | Pathogenic | 4,2 | 33% |
| 297 | FALSE | *BRCA2* | c.24_25insA | Pathogenic | 655, 89 | 12% |
| 110 | FALSE | *BRCA2* | c.2588del^a^ | Pathogenic | 26,13 | 33% |
| 332 | FALSE | *BRCA2* | c.36del^a^ | Pathogenic | 24,16 | 40% |
| 62 | FALSE | *BRCA2* | c.4284del^a^ | Pathogenic | 6,5 | 45% |
| 223 | FALSE | *BRCA2* | c.4361_4362insGA | Pathogenic | 6,2 | 25% |
| 284 | FALSE | *BRCA2* | c.5297del^a^ | Pathogenic | 9,3 | 25% |
| 332 | FALSE | *BRCA2* | c.7667del | Pathogenic | 13,3 | 19% |
| 110 | FALSE | *BRCA2* | c.8940del | Pathogenic | 10,8 | 44% |
| 168 | FALSE | *CDH1* | c.164-1G>T | Likely Pathogenic | 83,21 | 20% |
| 36 | FALSE | *MSH6* | c.1804_1805insA^a^ | Pathogenic | 238,47 | 16% |
| 147 | FALSE | *MSH6* | c.1804_1805insA^a^ | Pathogenic | 247,37 | 13% |
| 369 | FALSE | *MSH6* | c.1804_1805insA^a^ | Pathogenic | 285,46 | 14% |
| 116 | FALSE | *PALB2* | c.839del^a^ | Pathogenic | 609, 148 | 20% |
| 147 | FALSE | *PALB2* | c.839del^a^ | Pathogenic | 621, 126 | 17% |
| 297 | FALSE | *PALB2* | c.839del^a^ | Pathogenic | 432, 104 | 19% |
| 386 | FALSE | *PTEN* | c.821del^a^ | Pathogenic | 28,9 | 24% |
| 334 | FALSE | *TP53* | c.832C>G | Likely Pathogenic | 896, 140 | 14% |
|  |  |  |  |  | **Average MAF:** | **20%** |
| ^a^Homopolymer region  *^b^BRCA1* and *BRCA2* variants classified using the ENIGMA guidelines. Other gene variants classified using ACMG/AMP guidelines. | | | | | | |

Supplementary Table 3. Variants of unknown significance identified in 367 female breast cancer patients.

| Gene | Variant | Variant type | Predicted to alter splicing (MaxEntScan) | Classification | Rationale |
| --- | --- | --- | --- | --- | --- |
| *BRCA1* | c.626C>T | Missense | No | Uncertain | Insufficient evidence |
| *BRCA1* | c.1644T>G | Missense | No | Uncertain | Insufficient evidence |
| *BRCA1* | c.2613_2614delinsTG | Missense | No | Uncertain | Insufficient evidence |
| *BRCA1* | c.3068T>C | Missense | Yes | Uncertain | Insufficient evidence |
| *BRCA1* | c.3379T>C | Missense | No | Uncertain | Insufficient evidence |
| *BRCA1* | c.4096+3A>G | Splice Region | Yes | Uncertain | Insufficient evidence |
| *BRCA1* | c.4253T>C | Missense | No | Uncertain | Insufficient evidence |
| *BRCA1* | c.4357+1449G>A | Intron | No | Uncertain | Insufficient evidence |
| *BRCA1* | c.4675+1368G>A | Intron | No | Uncertain | Insufficient evidence |
| *BRCA2* | c.31T>C | Missense | No | Uncertain | Insufficient evidence |
| *BRCA2* | c.380C>A | Missense | No | Uncertain | Insufficient evidence |
| *BRCA2* | c.1626A>G | Missense | No | Uncertain | Insufficient evidence |
| *BRCA2* | c.2245A>G | Missense | No | Uncertain | Insufficient evidence |
| *BRCA2* | c.2661G>C | Missense | No | Uncertain | Insufficient evidence |
| *BRCA2* | c.2951A>G | Missense | No | Uncertain | Insufficient evidence |
| *BRCA2* | c.3853_3854delinsAG | Missense | No | Uncertain | Insufficient evidence |
| *BRCA2* | c.3994C>T | Missense | No | Uncertain | Insufficient evidence |
| *BRCA2* | c.3998A>G | Missense | No | Uncertain | Insufficient evidence |
| *BRCA2* | c.5800C>A | Missense | No | Uncertain | Insufficient evidence |
| *BRCA2* | c.5869A>G | Missense | Yes | Uncertain | Insufficient evidence |
| *BRCA2* | c.6220C>T | Missense | No | Uncertain | Insufficient evidence |
| *BRCA2* | c.6460T>C | Missense | No | Uncertain | Insufficient evidence |
| *BRCA2* | c.7130T>C | Missense | No | Uncertain | Insufficient evidence |
| *BRCA2* | c.8215G>A | Missense | No | Uncertain | Insufficient evidence |
| *BRCA2* | c.8855T>G | Missense | No | Uncertain | Insufficient evidence |
| *BRCA2* | c.887A>G | Missense | Yes | Uncertain | Insufficient evidence |
| *BRCA2* | c.9456G>T | Missense | No | Uncertain | Insufficient evidence |
| *BRCA2* | c.9984C>A | Missense | No | Uncertain | Insufficient evidence |
| *BRCA2* | c.10096A>G | Missense | Yes | Uncertain | Insufficient evidence |
| *CDH1* | c.387+5G>A | Splice Region | No | Uncertain | Insufficient evidence |
| *CDH1* | c.2048T>C | Missense | Yes | Uncertain | Insufficient evidence |
| *PALB2* | c.2087C>T | Missense | No | Uncertain | Insufficient evidence |
| *PALB2* | c.2200A>T | Missense | No | Uncertain | Insufficient evidence |
| *PALB2* | c.2704G>A | Missense | No | Uncertain | Insufficient evidence |
| *PALB2* | c.2708C>T | Missense | No | Uncertain | Insufficient evidence |
| *PALB2* | c.2816T>G | Missense | No | Uncertain | Insufficient evidence |
| *PALB2* | c.3007A>C | Missense | No | Uncertain | Insufficient evidence |
| *PALB2* | c.3351-6T>C | Splice Region | No | Uncertain | Insufficient evidence |
| *PALB2* | c.3495_3497delinsCTT | Missense | No | Uncertain | Insufficient evidence |
| *TP53* | c.993+234G>A | Intron | No | Uncertain | Insufficient evidence |
| *TP53* | c.993+310G>A | Intron | No | Uncertain | Insufficient evidence |

Supplementary Table 4. Characteristics of study cohort in Māori and non-Māori.

|  |  | **Maori** | | **Non-Maori** | | *OR [95% CI] P* |
| --- | --- | --- | --- | --- | --- | --- |
|  |  | **n=22** | | **n=345** | |  |
| **Average Age at Diagnosis (years)** |  | 57.9 | | 63 | | 5.15 [-0.36,10.66] 0.067 |
| **Grade** | **1** | 2 |  | 42 |  | 0.52 [0.21,1.28] 0.19 |
|  | **2** | 6 |  | 138 |  |  |
|  | **3** | 14 |  | 165 |  |  |
| **Estrogen Receptor** | **Pos** | 17 |  | 287 |  | 0.65 [0.23, 1.84] 0.38 |
|  | **Neg** | 5 |  | 55 |  |  |
|  | **N/A** | 0 |  | 3 |  |  |
| **Progesterone Receptor** | **Pos** | 18 |  | 253 |  | 1.54 [0.51,4.70] 0.61 |
|  | **Neg** | 4 |  | 87 |  |  |
|  | **N/A** | 0 |  | 5 |  |  |
| **HER2** | **Pos** | 5 |  | 43 |  | 1.75 [0.60,5.07] 0.35 |
|  | **Neg** | 15 |  | 226 |  |  |
|  | **Equivocal** | 1 |  | 31 |  |  |
|  | **N/A** | 1 |  | 45 |  |  |
| **Age diagnosed** | **<50 years** | 5 |  | 72 |  | 1.11 [0.31,3.29] 0.79 |
|  | **>50 years** | 17 |  | 273 |  |  |

Counts of samples in each category with odds ratios, 95% confidence intervals and Fisher exact tests between carriers and non-carriers with at least 1 observation in each cell. Grade has been collapsed to grade 3 vs grades 1 or 2.

Abbreviations: HER2, Human Epidermal growth factor Receptor 2 protein.
